# Sex: What Is the Big Deal? Exploring Individuals’ with Intellectual Disabilities Experiences with Sex Education

**DOI:** 10.1177/10497323211057090

**Published:** 2021-12-20

**Authors:** Rachelle Hole, Leyton Schnellert, Gloria Cantle

**Affiliations:** 1 UBC Okanagan School of Social Work, 97950 UBC Canadian Institute for Inclusion and Citizenship, Kelowna, BC, Canada; 2 8166 University of British Columbia, Vancouver, BC, Canada

**Keywords:** developmental disability < disability, disabled persons, sexuality, sexual health, qualitative

## Abstract

This article offers perspectives shared by self-advocates in the first phase of a community-based participatory research project untaken to address barriers that individuals with intellectual disabilities face with respect to sexual health knowledge. Using descriptive qualitative methods, we interviewed 19 individuals with intellectual disability about their experiences and knowledge related to sexual health. The research question guiding this project was: What are self-advocates’ with intellectual disabilities experiences learning about sexual health and sexuality? The findings highlight that participants faced barriers and lack of access to sexual health education, and while they learned about sexual health through formal sexual health education, frequently this knowledge came through lived experience. Finally, the findings underscore that participants knew what they wanted with respect to sexual health education and offered recommendations. The importance of accessible sexual health education for self-advocates that supports their rights and desires to express their sexuality and sexual agency is highlighted.

## Introduction

Sexual expression, including the right to develop relationships and access sexual health education, is an inherent human right. The World Health Organization specifies, “sexual health requires a positive and respectful approach to sexuality and sexual relationships, as well as the possibility of having pleasurable and safe sexual experiences” (2015, p. 5). The right to exercise sexuality includes the right to love and be loved in relationships with other people. Yet, people with intellectual disabilities (ID) often face discrimination and stigma when expressing their sexuality and face barriers when it comes to sexual health education (Abbott 2013, 2015; Wilkinson et al., 2015). This article centers the voices of self-advocates with ID regarding barriers to sexual health knowledge, sexual expression, and sexuality.

### Theoretical Lens

Our work is explicitly informed by critical disability studies (CDS). Such an orientation takes a critical approach to disability rights. “It foregrounds the experiences and perspectives of people with disabilities, maintaining that disability is a political and cultural identity, not simply a medical condition” (Dolmage, 2017, p.10). Rather than viewing disability as something that disqualifies or stigmatizes, CDS affirms disability as a valued part of human diversity (Erevelles, 2014, p.2). In our program of research, we seek to disrupt ableism to advance and promote the citizenship of individuals with ID, who we refer to as self-advocates (SA). The term “self-advocate” positions individuals with ID as self-determining and acting with agency (Anderson & Bigby, 2017; Schnellert et al., 2021).

Such a stance aligns with Nussbaum’s approach to justice as articulated by Kulick and Rydström (2015) that ensures “fundamental human entitlements that allow people to live with dignity and develop their capability to exist and flourish in the world” (p. 278). Thus, with respect to sexuality, we see it as imperative to frame the issue of self-advocates’ sexuality in terms of agency and independence, while acknowledging that some intellectual impairments may at times entail interdependency that requires supportive affirmative actions that support individuals to develop his/her/their capabilities to the fullest extent possible (Kulick & Rydström, 2015, p. 281).

### Literature Review

#### Self-Advocates as Sexual Beings

Individuals with a label of ID are often framed as lacking cognitive capacity for sexuality, rather than recognized as sexual agents with diverse sexualities. Such a stance results from sexual ableism (Gill, 2015). Sexual ableism produces ID as a characteristic that disqualifies one from sexual agency and it negates the rights of these individuals as sexual beings (McCarthy et al., 2020). When it comes to sexuality for people with ID, they are often constructed as asexual, sexually inactive, or conversely, sexually deviant (Kulick & Rydström, 2015; Shakespeare, 2000). These myths and stereotypes endure (Gill, 2015) and create obstacles and stigma around sexuality for individuals with ID; putting them at greater risk for sexual exploitation, disease, and/or mental health issues (Irvine, 2005). Contrary to these stereotypes, individuals with ID are neither asexual (Abbott & Howarth, 2007; Rohleder & Swartz, 2009) nor sexually inactive (Azzopardi-Lane & Callus, 2015).

People with ID desire meaningful relationships, which can range from friendships to sexual intimacy (Brown & McCann, 2018; Kelly et al., 2009). In research by Healy and colleagues (2009), participants with ID understood important components of relationships, including trust, mutuality, and companionship. Gil-Llario et al. (2018) found 84% of the 360 participants with ID had been in a sexual relationship and 87% indicated they currently would like to be in a relationship, highlighting the importance of relationships for this population. Moreover, sexual pleasure is an important aspect of relationships and sexuality for people with ID. In Turner and Crane (2016), participants described a diversity of experiences including “kissing, having a crush, sexual talk, ‘lovemaking’, anal intercourse, ‘playing doctor’, marriage, dating, expressing ‘I love you’, fantasy, masturbation, fellatio, vibrators, and pornography” (p. 687).

#### Barriers to Relationships

Individuals with ID face barriers to relationships (Gill, 2015). One frequently reported barrier is caregivers—family members and/or support workers—who act as gatekeepers constraining the relationships of people with ID that they support (Abbott, 2013; Azzopardi-Lane & Callus, 2015; Bernet & Ogletree, 2013). Researchers report instances where staff or parents chastised participants for physical intimacy, teased them when discussing sexual topics, and where participants’ partners were not allowed to stay overnight (Antaki et al., 2007; Kelly et al., 2009; Hollomotz & The Speakup Committee, 2009; Turner & Crane, 2016). Surveillance and suppression of relationships and sexuality can put adults with ID at risk and can lead to risky behavior. Hollomotz and The Speakup Committee (2009) found that participants in their study resorted to being sexually active in public or semi-private places due to the lack of privacy in residential care settings and the restrictive approaches of staff.

People with ID indicate they desire support from caregivers in their relationships (Bane et al., 2012). Although support staff are more likely to discuss sexuality and relationships with people with ID than family members, some staff are uncomfortable with this topic due to inadequate training on this issue, conflicting parental wishes, lack of organizational policies, and lack of confidence (Evans et al., 2009). To complicate matters, sexual health education for individuals with ID is lacking.

#### Sexual Health

The importance of sexual health education for people with ID cannot be overlooked, as insufficient knowledge may increase the risk of sexual abuse and sexually transmitted diseases (McDaniels & Fleming, 2016). The risk of sexual abuse is higher for people with ID (Fisher, Bair, Currey, et al., 2016). Exact rates vary, but Tomsa and colleagues (2021) reported that one in three adults with ID experience sexual abuse as adults. Another review reports 7–34% will experience sexual abuse (Smit, Scheffers, Emck et al., 2019). People with ID have varying, degrees of sexual knowledge, but overall appear to have a limited understanding of sexuality and relationships (Jahoda & Pownall, 2014). Knowledge of sexually transmitted diseases and contraception is the most lacking, while parts of the body/physical traits were the most understood concepts (Borawska-Charko et al., 2017). For example, one study found that young adults with mild/moderate ID are 50% more likely to have unsafe sex (not using contraception or precautions) than their peers without ID (Baines et al., 2018).

There is a paucity of research regarding effective and empirically validated sexual health curricula for people with ID (McDaniels & Fleming, 2016). Sexual health education is often “taught reactively” after problems arise, rather than proactively (Abbott & Burns, 2007; Schaafsma et al., 2015). Further research is needed to understand the frequency of safe/unsafe sexual practices and the different factors that impact the sexual health knowledge of this population (Borawska-Charko et al., 2017). Critically, more information is needed on the effects of stigma and social isolation on the sexual experiences of people with ID (Borawska-Charko et al., 2017).

#### Sexual Identity and Diversity

Education about positive sexual expression and issues relating to sexual diversity needs to increase through sexual health education (Murphy et al., 2007; Wells et al., 2014). However, social discourse and sexual health education are predominantly heteronormative when it comes to the identities of person with ID. Löfgren-Mårtenson (2009) found that caregivers identified homosexual behaviors as “experimentation” rather than as indicators of LGBTQI+ identity. Relatedly, Abbott and Burns (2007) highlight that the LGBTQI+ community has been “less than welcoming” of people with ID, recommending more targeted resources for this segment of the LGBTQI+ community.

## Methods

In light of the above findings and research gaps, we undertook a qualitative study to explore experiences of sexual health education among adults with ID. The research question guiding this project was: *What are self-advocates’ with intellectual disabilities experiences learning about sexual health and sexuality?* Qualitative description (QD) (Hyejin et al., 2016; Sandelowski, 2000, 2010) was used to ascertain the perspectives and experiences of 19 adults with ID regarding sexual health knowledge, education, and relationships. QD is especially relevant for research projects “aiming to gain firsthand knowledge” of individuals with lived experience regarding a particular topic (Hyejin et al., 2016, p. 24)—in this case self-advocates’ lived experience. Moreover, Lloyd, Gatherer and Kalsy (2006) assert that qualitative research with people with expressive language difficulties, such as people with ID, can serve as a means of validation and empowerment. Another advantage of QD is its suitability to projects where time is limited (Neergaard et al., 2009); such was the case in this study which was the first phase of a 3-year participatory theater-based research project. Ethics approval was granted by the University of British Columbia Behavioural Research Ethics Board [H17-00664].

### Sampling and Recruitment

Using convenience sampling, individuals were recruited through local community living organizations in the Lower Mainland and the Central Interior of British Columbia, Canada. Recruitment flyers were distributed and interested individuals were invited to contact the lead researcher or the research assistant for more information; alternatively, potential participants signed a consent to contact form, and the research assistant followed up directly to explain the project and answer any questions. If the individual expressed interest in participating, an interview time was set for a later date. Inclusion criteria were as follows: (a) an individual who receives, or who is eligible to receive, services from the Crown Agency responsible for community living supports and services in BC; (b) an individual over the age of 18; and (c) an individual consenting to participate. Nineteen individuals met the inclusion criteria and agreed to participate. The final sample composition was determined by the scope of the study and the quality of the data (Morse, 2000), and recommended adequacy standards for QD studies (Bradshaw et al., 2017).

With respect to consent, we agree that “adult persons with intellectual disability should be assumed capable of providing informed consent, unless it has been established otherwise” (Dalton & McVilley, 2004, p. 62). This position is in line with Article 12 of the UN Convention of the Rights of Persons with Disabilities (2006) and the D.A.I. Judgement of the Supreme Court of Canada (R. v. D.A.I., 2012 SCC 5, [2012] 1 S.C.R. 149). That said, safeguards were put in place regarding the process of consent: the use of a plain language consent form and the use of interviewers with many years of experience working with individuals with ID. Of note, all three authors have extensive experience working in the area of intellectual disability (education, research, and lived experience) ranging from 10 to 35 years. Interviewers confirmed both verbal and written consent from participants prior to commencing the interview. In line with the above positions, all participants were recognized as capable of providing consent.

### Data collection

Semi-structured individual interviews were conducted with participants. With participants’ consent, all interviews were digitally recorded and transcribed verbatim, except three interviews when the participants declined to have the interview recorded. In these instances, detailed field notes were taken. Interview topics included an exploration of the individuals’ experiences learning about sexual health and sexuality, including content, perceived gaps in sexual education for both K-12 and adults, and advice/suggestions for sexual health educators. Topics for the interviews were identified in consultation with a sexual health educator working with individuals with ID. As we progressed with the interviews and early data analysis, we focused our inquiry looking for variations in participants’ accounts of their experiences. Data collection stopped at 19 when the team determined saturation had been reached.

### Data analysis

Qualitative content analysis, a common strategy for analysis in QD studies (Hyejin et al., 2016) informed the analytic process. In terms of analysis, the aim of QD is “a rich, straight description of an experience or event” (Neergaard et al., 2009, p. 24). Thus, content analysis allowed the researchers “to stay close to the data, with minimal transformation during analysis. Such interpretation is low inference” (p. 24).

Data analysis began immediately after interviews were completed. The interviews were transcribed verbatim. Using Nvivo 11 to facilitate data analysis an initial coding framework was developed. QD analytic strategies were implemented: coding of interview data; recording insights and reflections; sorting through the data to identify similar important features; looking for commonalities and differences among the data for further consideration and analysis; and deciding on categories that hold true for the data (Neergaard et al., 2009). To address validity, categories were verified through re-reading the data and keeping an open mind about the connections of meaning that the data revealed (Creswell & Miller, 2000). Regular meetings were held with research team members to address analytic validity: using NVivo, coding reports were run, and reviewed and discussed by team members allowing for re-reading, reviewing, and refining of the findings.

In line with descriptive studies (Bagwell-Gray, 2018; Pope et al., 2018), presentation of study findings are comprehensive descriptive accounts of the data (Hyejin et al., 2016). As Neergaard et al. (2009) describe, “the final product of QD is a description of informants’ experiences in a language similar to the informants’ own language” (p. 2). The findings are supported by verbatim quotes from participants.

### Participants

Nineteen individuals with ID participated in this study. Eleven of the individuals were female, seven were male, and the demographic information for one was missing. Fourteen participants lived in the Lower Mainland of BC (near Vancouver) and five resided in the Central Interior. Two of the participants were currently married to each other for 19 years; one had been married in the past; and several other interviewees were currently or had been in relationships. The age range of the participants was 18–51 years.

### Findings

Participants’ responses to interview questions regarding sexual health education described several common elements and exposed gaps in their formal learning. The first finding focuses on participant descriptions that indicated barriers and lack of access to sexual health education. The second finding presents SAs’ descriptions of knowledge derived through formal sexual health education and lived experience. Finally, the third finding focuses on participants’ recommendations for future sexual health education. These findings center self-advocates’ (SAs) voices and perspectives regarding the sexual health knowledge they want and need in order to experience positive sexuality.

### Lack of Access

SAs articulated several barriers that they experienced with respect to accessing sexual health knowledge and information in any context, including with their families. The two most common issues were adult discomfort and gatekeeping, and lack of content related to sexual diversity.

#### Discomfort

SAs’ responses pointed to adult discomfort regarding the sex lives of individuals with ID. Participants expressed that parents and caregivers were uncomfortable talking about sex. One individual described talking to her friends about sex rather than adults:

I’d talk about it with my friends or whatever. Not like obviously, but we can even make jokes or whatever. But yeah. Nobody adult-wise really… Like, especially my auntie. Because I grew up with my auntie, not my mom, and she never really talked to me. It was like this big scary thing. … When I asked questions about it, my auntie would just shut me right down. She’d like, “That’s inappropriate.” … she made it seem like it was such a bad thing. She made it seem like it was wrong that I was asking those questions.

Another participant concluded from his own experience that sexuality can be a challenging topic to talk to children about before high school: “It’s kind of one of those things when you’re a kid that people really don’t want to tell you until high school or later on. […] It’s one of those difficult things to talk to your kids about.”

Several participants expressed frustration with caregivers’ unwillingness to discuss sex and sexuality. One person pointed out, “You gotta have an open dialogue.” Another individual described a conversation with her mother about sex, saying, “She got the book from there ‘cuz there was a whole course on how to talk to your child about it. It’s like, dude, just talk about it.”

Gatekeeping and discomfort significantly limited access to sexual health knowledge for individuals with ID. As explained above informal familial discussions about sex were often strained and/or one-time events. Interviews also revealed a lack of attention to sexual diversity in formal and informal education for participants.

#### Lack of Content Related to Sexual Diversity

None of the self-advocates described learning about LGBTQ+ relationships in formal sex education. For example, two participants were unaware of the term “sexual diversity.” When one of the individuals was asked if they had any education about sexual diversity, they replied, “Nope… I don’t know what that means.” When it was explained that this referred to people who are LGBTQ+, a participant replied that she knew about sexual diversity because of TV: “Because in the TV, they’ll talk about the gay. Also, they’re wearing like earing out, outside. There is tattoo, so they do it as well because they want to be a gay.” Four other participants described learning about LGBTQ+ relationships through television:

I have been watching a lot of TV about that lately. […] And lately I heard that they actually finally, I think it was with the government or something about being married or something. […] They got their rights back.

Overall, participants were interested in learning about sexual diversity and were supportive of the LGBTQ+ community. For instance, one individual felt sexual health education should be more “About diversity. That some people want to be gay and that’s okay with me if that’s okay with them.” However, two participants expressed that they did not support LGBTQ+ relationships. Stigma about sexual diversity had personally impacted a self-advocate who learned, “Gay was bad and going to gay clubs was bad.” This individual went on to explain, “A female doctor called me out for having an STI because being gay was bad.” Such stigmatizing experiences and attitudes offer little opportunity for prosocial sexual identity development. Heteronormative perspectives and assumptions about individuals with ID seemingly erased sexual diversity from sexual health education. This meant that they faced increased barriers accessing information based on their individual circumstances.

Many of the interviewees were not familiar with the concepts or language of sexual diversity. Whether discomfort or gatekeeping, this silence disempowered participants in this study. Learning about LGBTQ+ identities and sex through such means as television and/or stigmatizing comments limited their opportunities for meaning-making and healthy sexual identity exploration.

### Knowledge derived through formal sexual health education and lived experience

In regards to formal sexual health education, SAs overwhelmingly reported the strongest memories of content as focused on sexually transmitted infections, condom use, and pregnancy. The second area emphasized by the participants related to information shared about human anatomy. A third content area described by SAs was about the importance of safety and consent. This was in contrast with what they deemed to be their most significant experiential learning which was/is relationship oriented.

#### STIs and Pregnancy

Across interviews SAs reported that their sexual health education emphasized sexually transmitted infections, condom use, and pregnancy. This emphasis left them with internalized messages that focused on risks and danger. What they heard is that they should mitigate the dangers. An individual described, “The first one I remember was the basic part about HIV and stuff like that and the pictures. […] And to be aware of that […] if you’re going out with somebody you never know, like, if they have it or not.” Similarly, another participant stated, “I remember birth control was a big thing.” One person described learning to put a condom on a banana in school and later stated, “I know better. Because if I didn’t bring condoms there’d [be] no sexuality.”

#### Human Anatomy

Another subject strongly emphasized in formal education was human anatomy and sexual reproduction such as a focus on body parts and the mechanics of sex. This limited the breadth of what was learned, shutting down access to richer, more nuanced explorations of sex and sexuality. A participant articulated that her class focused on the male and female organs: “When I first started learning it was about,‘Oh, here’s a penis and here’s a vagina - ya go together, ya do things. Yep, basically, that’s basically what we learned about.” Similarly, an individual shared that his sexual health education was limited and attended to, “Just how [babies are] made and why periods were done.[…] And the ins and outs of the body parts… Just like the privates like … the organs and what attaches it all together and makes it all work.”

#### Safety

Safety and consent was another central topic in participants’ formal education. However, SAs’ sexual education might best be described as mechanistic and lacking attention to positive sexuality such as self-efficacy, sexual pleasure, or sexual satisfaction. One person explained, *“*Well, I guess it was about sex, but it was just like, it’s not appropriate for somebody to touch you when you don’t wanna be touched, like your private areas. I just remember them giving us that talk.” Similarly, another participant described the conversations she had with her mother as focusing on the same topics:

[We talked] about being safe, about sex in a safe way. If you don’t want to have sex you can always remove yourself, or tell that person how you feel, or maybe just walk away if that person is still bothering you about it. Or tell someone… It might lead to a rape or assault, you never know.

Conversations about safety emphasized the risks of sex. This was reflected in several participants’ understanding of sexual intimacy. For instance, an individual learned, “Don’t have sex. If you don’t have sex then you can’t catch stuff but people still do it sometimes.” Another self-advocate also felt that having sex was a “gamble” in that “it’s good because you can have like babies, but then again the chances of getting HIV and STDs and all these other stuff [is bad].”

#### Relationships

SAs deemed relationships to be an important part of their learning. Participants appeared to learn about relationships through experience rather than by formal education. For example, during the interview when a participant was asked whether he learned about relationships in sex education, an individual replied that he had been in four or five relationships himself but did not learn about relationships formally.

When participants were asked about what they had learned through experience beyond their formal and informal sexual education during their teens, one stated, “Relationships. How can you have sexuality without relationships?” Another person expressed that he learned “love and friendship” are important and wanted to find someone he could “trust and go out with.” Moreover, participants’ feedback regarding what’s important to know and learn highlighted qualities of respect and parity within relationships. An individual explained,

The important things [are] that you should have dignity and respect towards the opposite sex. And, equality too. The reason for that is because…there’s a saying behind it…that how do you want to be treated? I would want to be treated as fairly and equally as possible, with dignity, equality, and respect. That’s how I wanna be treated. That’s how I treat others too. No matter what gender they are.

Participants described nuanced learning through lived experience that they used to make decisions as they navigated desire, sexual activity, and choice making. For example, one person stated, “If I had a boyfriend, I would have sex but… Yeah. Not [at] the wrong time.” Similarly, another individual desired intimacy with a friend but was waiting until they knew each other more, explaining,

I told him I have a son and he ask[ed] me, “How?” I say, “I sleep with my husband.” He asked me, “Would you like to do it with me?” I said, “Yes. Not right now. I want us to, like slowly, slowly because we just [started to] know each other.

Participants deemed knowledge about relationships, desire, and intimacy to be their most significant experiential learning and wanted formal sex education to address this. Across interviews, it was evident that self-advocates desired loving relationships within which they could engage in intimate conversations, have mutually satisfying sexual encounters, and build a life together over time. However, for those who had achieved these goals, almost all of the knowledge that they had acquired was through lived experience and that education that supported this learning was incidental and/or contextual. Lack of content related to relationships in their sexual health education raises questions about the assumptions of educators, marginalizing consequences for self-advocates, and significant inequities.

### Recommendations: Centering Self-Advocates in Sexual Health Education

Over the past few decades, the self-advocacy movement is flourishing in numerous jurisdictions, including Canada. The self-advocacy movement emphasizes individuals with ID (self-advocates) rights and abilities to speak out, to have a say, and to experience empowerment. Participants in the current study demonstrated their desires for self-advocacy by highlighting ways in which their voices should be centered in sexual health education. For example, and in line with previous research (see Discussion) a more relational approach to sexual health education was recommended by participants; A self-advocate suggested educators should work with individuals with ID and their partners: “Yes, work with couples, see if their relationships are doing well. . . It’s like do you take birth control pills? Or do you have something special in your tummy so you don’t get pregnant?” There were several additional messages from self-advocates that we would like to highlight.

#### Expert Sexual Health Educators

Participants wanted sexual health education to be taught by a trained professional, or as one individual put it, someone who “knows what they are talking about.” This perspective was echoed by another person who stated, “I would say a professional to be honest because they need to - a professional or a health care person. That’d be awesome if they had both.” Self-advocates distinguished between being taught by a professional compared to a teacher. A individual described,

It should have been more of - not a teacher - but a person from the health unit coming to talk because they know more about sexual things too. I felt like the teacher didn’t have much - they were good - but not too good.

However, another participant articulated that people with diverse abilities should also be instructors in sexual health education, not just professionals.

They should have a self-advocate involved in it too. Because a lot of people who have a diverse ability don’t really wanna go to a doctor a lot of the time. They don’t wanna confront the doctor because the doctor sometimes makes us feel like we’re crap and we’re not worth being cared for. […] I feel like, we as people need to have more, like, not have a one-way mind, but have a person with diverse abilities, have somebody with those experiences talking about their experience. And then I feel like it would make it better because it would draw out more of the experience, and less of, “Oh, here’s a doctor, oh, we should listen to this person.”

Overall, self-advocates emphasized that a sexual health educator be informed and be able to deliver material/content in a manner that allowed the audience to feel safe and comfortable.

#### Accessibility

Participants indicated that sexual health education was often targeted to neurotypical students and was not accessible for people with intellectual disabilities, stating,

They presented it through slides and they basically made it so that it was comprehensible to a person without a disability. They made it so that a typical person would understand it. They didn’t make it so that I would understand it. And, a lot of the times I felt that I basically was in the dark about a lot of the sexual stuff and relationships.

Three self-advocates had difficulty understanding information in textual format. One individual described an instance where sexual health information was on a poster, but he pointed out, “That wasn’t helpful if you didn’t know how to read.” Another recommended that “if people know how to read, if they have it on paper, but people that don’t know how to read should have pictures. […] It makes it fair so they don’t feel left out.” Four self-advocates suggested pictures would improve sexual health education. Other participants encouraged a hands-on approach to learning, explaining,

I remember putting condoms on. They would be throwing condoms at all of us. I remember that. But that was more … I don’t know. For some reason, condoms just make it all fun. So, if you’re going into high school, bring lots of condoms to make it fun and colored ones. […] I remember grabbing like 100 of those [condoms].

Self-advocates also recommended that videos and the internet, such as social media, should be used to provide information. An individual said she had seen sexual health cartoons, which explained “menstruation, sex and stuff, where babies come from, and actually how babies were made. It was so neat.” Participants were flexible as to what kind of videos should be made about sexual health. When a self-advocate was asked whether she thought videos should be animated, cartoons, drawings, or dialogue-based she responded, “altogether, I would say. […] Nothing in particular. I enjoy a bit of all.”

#### Disability-Specific Representation and Content

Self-advocates wanted a greater representation of people with diverse abilities within the curriculum. An individual described that her sexual health class was “just about the normal body, not disabilities, but just about the normal body. And, how your body changes.” This lack of representation made her feel “discriminated [against]… ‘cuz I’m a person with disabilities.” She went on to add, “I think it should be more including. It should be different because we are people with disabilities and they should teach about people with disabilities and sexuality. Not [just the] ‘normal’ body.” This sentiment was echoed by another participant:

I definitely think they could have made it a little bit more diverse. […] Then it’s like, there’s a lot missing from that picture because a lot of us need a little bit of, whether it’s physical help, or whatever, a lot of us need help to maybe get on top of that person.

#### Ongoing Adult Sexual Health Education

Self-advocates desired further sexual health education after high school. One person explained:

Well after, when I left high school, I was actually starting to forget like everything. And I was like thinking in my head, like okay be a lot more better Andrew, learn it back all over again. Especially after starting a relationship out here. And I was looking for somebody who I could trust and someone who I could go out with. I was only 24 at the time.

Similarly, another participant felt, “You need to have that [sexual health information] in the community too, beyond the walls of the educational institution because I don’t want people to fall into the cracks.” He explained this education would be different from high school:

It would be a refresher. Growing up as a teenager, they probably think, yes, thumbs up, you know - how teenagers would react to it. But, then if you have a refresher course after high school, they might stop and think.

The benefits of adult education were further expressed by a person who said, “When we’re in adulthood and teenage years, for myself, I think I would take it more seriously.” However, she specified that adult education should be “not something that’s boring but something that’s actually exciting for people.”

Participants were generous, for the most part, about the efforts of educators, but also felt that their perspective could alter current practice. Self-advocates wanted to influence sexual education, whether that be informing who presents the material, the kinds of material included, and when sex education should be available. Self-advocate participants advocated for their voice to be heard and communicated that their input would improve sexual health knowledge for other self-advocates by making the material more relevant and accessible.

## Discussion

The research question, *What are self-advocates’ with intellectual disability experiences learning about sexual health and sexuality?* was used to guide this study. Qualitative description (QD) assisted us to center the perspectives and experiences of adults with ID in line with our critical disability studies theoretical framework. Participant descriptions illustrated barriers and lack of access to sexual health education. There were notable differences in descriptions of knowledge derived through formal sexual health education versus lived experience. Finally, participants offered recommendations for future sexual health education highlighting, in particular, the need for disability-specific representation and content. These findings center self-advocates’ voices and perspectives regarding the sexual health knowledge they want and need in order to experience positive sexuality.

In line with other research, these findings demonstrate that people with ID desire relationships, want to develop relationship skills, and are eager to learn more about this topic (Azzopardi-Lane & Callus, 2015; Brown & McCann, 2018; Friedman et al., 2014). Kulick and Rydström (2015) write, “each human life should have the opportunity to develop and explore her or his erotic awareness and capacities and to be given the possibility of extending herself or himself in ways that engage sensations, activities, and relationships that can provide pleasure, comfort, self-respect, and satisfaction” (p. 292). Many self-advocates in this study described lived experiences with sex, sexuality, and relationships. What they wanted was access to education that as Kulick and Rydström (2015) explain, “raise[s] the topic of sex and talk [s] about it in ways that highlight [s] sexual expression and sexual pleasure (of saying “yes” to sex) instead of always framing sex exclusively in terms of protection and abuse (of only saying “no”)” (p. 292–293). Likewise, young adults without ID also prioritized relationships in their sexual conversations. Faulkner and Lannutti (2010) found that young adults’ conversations were more focused on sexual pleasure and relationships than sexual health and whether these conversations where satisfying impacted the quality of the relationship. Therefore, a more relationally focused curriculum could be beneficial for all young adults, including self-advocates.

Participants in this study navigated relationship issues with their partners but received little, if any, education about this topic. They recognized the importance of communication in healthy relationships and stated that support from professionals could help overcome issues in these areas. In contrast, participants’ sexual health education placed an overwhelming emphasis on biology, safety, and consent. These findings are similar to Frawley and Wilson (2016), whose participants received the main message that sex was something to be worried about or frightened of but did not learn information about positive sexuality. We echo recommendations from other studies, asserting that while education about basic biology and abuse/exploitation education is highly important, there needs to be greater emphasis on sexual diversity, relationships, communication, pleasure, and other positive areas of sexuality (Brown & McCann, 2018; Chrastina & Večeřová, 2020; Kelly et al., 2009; Turner & Crane, 2016). Further research could explore one participant’s suggestion to work with couples, studying whether couples education with people with ID improves relationship dynamics and sexual health knowledge. Neuman and Reiter (2017) found that people with ID who were in intimate relationships scored higher on quality of life measures, had a more positive self-concept, and a greater sense of independence than participants in close friendships.

Erasure of sexual diversity as a consequence of the mechanistic and limited scope of sexual health education is an issue raised by this research. Sexual health education relating to LGBTQI+ relationships was overlooked; none of the participants described learning about this subject in formal settings. Importantly, Friedman et al. (2014) found that accessible information about sexual diversity is needed for self-advocates to make informed decisions about being in LGBTQI+ relationships and to be knowledgeable about all the options they have to choose from. Moreover, people with ID who identify as LGBTQI+ face significant barriers to having relationships due to discrimination, as well as service providers/caregivers failing to support them in this area (Abbott & Burns, 2007; Abbott, 2015). Sexual health educators could help reduce these barriers through LGBTQI+ support groups. In fact, Moreno and colleagues (2017) found that attending an LGBTQI+ support group helped participants with ID foster a sense of purpose, increase self-esteem, develop a sense of pride, and improve psychological well-being. Also, sexual health education needs to represent people with the intersecting identities of diverse sexual orientations and ID within the curriculum. People with ID have the right to engage in LGBTQI+ relationships and education should acknowledge, support, and promote these rights.

Our findings highlighted that parents and caregivers were uncomfortable discussing sexual health topics, whereas self-advocates desired an, *“open dialogue.”* Paternalistic attitudes predominate among caregivers regarding the sexuality of people with ID and caregivers tend to communicate that sex/sexuality is inappropriate (Kramers-Olen, 2016). Caregivers have also described a tension between empowering the sexuality of a person with ID and concerns about the risk of exploitation/abuse (Kramers-Olen, 2016; Wilkinson et al., 2015). Healy et al. (2009) argued that staff can discourage the relationships of people with ID due to unclear guidance, policies, and legislation. These findings illustrate the need for changes in agencies with respect to policy and practice in order to promote sexual empowerment. Finally, training may be a way to reduce the restrictive views of caregivers; Grieve et al. (2009) found that staff with more training on sexuality were more open to people with ID having relationships. Greater support from caregivers is essential, since a prohibitive and paternalistic approach creates undue obstacles for people with ID to live sexually fulfilled lives. Kulick and Rydström (2015) underscore the need to engage with self-advocates around their erotic feelings, curiosities, and desires. This means “understanding that individuals with disabilities, just like everyone else, need help and support in acquiring ways to comprehend and express their sexuality. This means cultivating an awareness of signs that might indicate an interest in or a curiosity about sexuality” (p. 292–203).

Finally, this study highlights areas to be explored in future research. Researchers could investigate the impact of sexual health education when taught by a professional and a self-advocate as compared to professionals only; involving self-advocates in sexual health education may help reduce inherent power dynamics and further promote sexual rights. In Friedman et al.’s (2014) study on sexual self-advocacy, participants asserted the need for professionals and caregivers to receive additional training on disability awareness and how to respectfully support the sexual needs of people with ID. Involving people with ID as educators would recognize their knowledge and experience as legitimate sources of information for others to learn from.

Participants in this study felt left out in sexual health education because their programs did not represent people with disabilities and were not adapted to their disability-related needs. Similar to other research, participants wanted more accessible information in the form of pictures, videos, hands-on learning, and social media (Friedman et al., 2014). Information should not just show “normal bodies,” but should express how people with a variety of disabilities can engage in sexual intimacy in pleasurable ways. Also, accessible materials should be developed in collaboration with self-advocates to further ensure that sexual health information is inclusive and effective for this population group. Despite the paucity of empirically supported sexual health curricula for people with ID (McDaniels & Fleming, 2016; McGuire & Bayley, 2011), one proven adaptation for practice is individually tailored sexual health education. In Dukes and McGuire (2009), participants involved in personally adapted curriculum improved their capacity to make decisions about sex and sexuality after receiving the education. However, this study did not have a control group and further quantitative research is needed to develop a stronger evidence base for sexual health curriculums for people with ID.

## Conclusion

These findings demonstrate that positive sexuality is often not promoted in sexual health education for individuals with ID. Participants desired attention to relationships as a central topic of and as an approach to sexual health education, yet learned mostly about safety, consent, and biology. Although these are vitally important topics, education should put more focus on the positive elements of sexuality, such as communication, intimacy, and pleasure. Furthermore, people with ID need to be represented in the curriculum, have greater access to accessible sexual health information, and be involved on a teaching level for professionals and individuals during sexual health education sessions. People with ID have the right to love and be loved, which includes sexual intimacy if they choose. Sexual health education needs to support and promote this right to sexual citizenship and agency.

## Supplemental Material

sj-pdf-1-qhr-10.1177_10497323211057090 – Supplemental Material for Sex: What Is the Big Deal? Exploring Individuals’ with Intellectual Disabilities Experiences with Sex Education Click here for additional data file. Supplemental Material, sj-pdf-1-qhr-10.1177_10497323211057090 for Sex: What Is the Big Deal? Exploring Individuals’ with Intellectual Disabilities Experiences with Sex Education by Rachelle Hole, Leyton Schnellert and Gloria Cantle in Qualitative Health Research
