# Supplementary material for: Sex: What Is the Big Deal? Exploring Individuals’ with Intellectual Disabilities Experiences with Sex Education
Source: Qual Health Res. 2021 Dec 20;32(3):453–64. doi: 10.1177/10497323211057090 (PMC8796054; doi:10.1177/10497323211057090)
Supplement: sj-pdf-1-qhr-10.1177_10497323211057090 – Supplemental Material for Sex: What Is the Big Deal? Exploring Individuals’ with Intellectual Disabilities Experiences with Sex Education [file sj-pdf-1-qhr-10.1177_10497323211057090.pdf]

Sex, Lies and Citizenship
Self Advocate Interview Guide

I) **Review** consent form

II) **Introduction:** Today I would like to talk with you about your experiences of sexual knowledge (learning about sexual relationships and your body)...

We want to know what your experiences have been like... what are the good things, what are the difficult (bad) things?

I want to learn about how and where adults with developmental disabilities get sexual knowledge. I am going to ask you some questions about your experiences about sexual knowledge. You do not have to answer my questions if you don't want to. You will not get in trouble. If you want to we can stop the interview at any time.

III) Topics and Probes:

a. Who told you about sex?:
    i. How do you know that person who talked to you about this?
    ii. Has more then one person talked to you about sexual education?
    iii. Are there other ways you learn about sex? (Books, Friends, Caregivers, the internet?)
    iv. Did you think they were comfortable/ nervous talking to you about sexual education?
        1. How could you tell?

b. The experience:
    i. What was positive (good) about learning about sexual education from (name of book, friend, caregiver, internet site)?
    ii. What message do you think they were trying to tell you about sexual education?
    iii. What was helpful?
    iv. What was not helpful?
    v. What could that person/those people have done differently?

c. The content: What kinds of things have you learned about sex and sexuality…?
    i. When you were young?
    ii. As a teenager?
    iii. As an adult?
    iv. Probe topics: e.g., body parts, safety, consent, pregnancy, STIs, sexual diversity/LGBTQ, other

d. Future directions
    i. What do you think someone should tell you (teach you) about sexual education?
    ii. Where would you like to learn about sexual education?
    i. Who would you like to get sexual education from?
    iii. What would you like to do different? If there is something that you would like to do different, what would that be? How would you make that happen?
